# Natriuretic peptide biomarkers in the imminent development of preeclampsia

**DOI:** 10.3389/fcvm.2023.1203516

**Published:** 2023-07-24

**Authors:** Stefanie Marek-Iannucci, Estefania Oliveros, Yevgeniy Brailovsky, Preethi Pirlamarla, Amanda Roman, Indranee N. Rajapreyar

**Affiliations:** ^1^ Advanced Heart Failure and Transplant Cardiology, Thomas Jefferson University, Philadelphia, PA, United States; ^2^ Temple Heart and Vascular Institute, Temple University, Philadelphia, PA, United States; ^3^ Advanced Heart Failure and Transplant Cardiology, Mount Sinai Hospital, New York, NY, United States; ^4^ Obstetrics and Gynecology, Maternal and Fetal Medicine, Thomas Jefferson University, Philadelphia, United States

**Keywords:** preeclampsia, NTproBNP, heart failure, peripartum cardiomyopathy, biomarker

## Abstract

Preeclampsia is the most common cause of morbidity and mortality in pregnancy, the incidence being significantly higher in low-income countries with reduced access to health care. Women with preeclampsia are at a higher risk of developing cardiovascular disease with a poorer long-term outcome. Early recognition and treatment are key to improving short- and long-term outcomes. Approximately 3%–5% of pregnant women will develop preeclampsia, with potentially fatal outcomes. Despite ongoing research, the exact pathophysiologic mechanism behind its development remains unclear. In this brief report, we describe the potential role of natriuretic peptides as biomarkers in the imminent development of preeclampsia. In a retrospective manner, we analyzed changes in the left ventricular ejection fraction and left atrial volume and increases in natriuretic peptide in correlation with the development of preeclampsia. We found that three out of four patients developed a significant increase in natriuretic peptide, which correlated with the development of preeclampsia and/or peripartum cardiomyopathy. Significant increases in natriuretic peptides around the time of delivery might be a marker for the imminent development of preeclampsia. Close monitoring of natriuretic peptide levels in the peripartum period could give important insight into the imminent development of preeclampsia in high-risk patients. Close follow-up in specialized cardio-obstetric clinics is highly recommended.

## Graphical Abstract

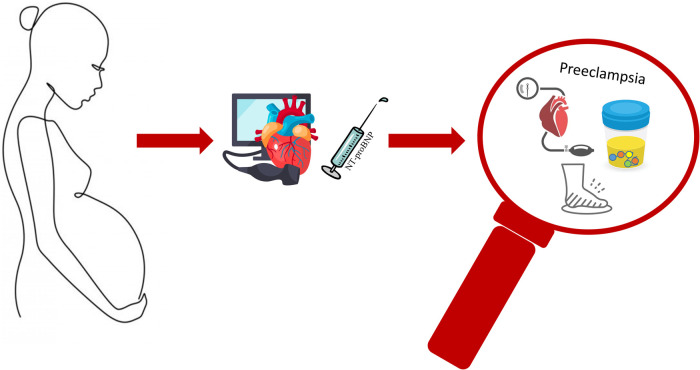

## Introduction

Preeclampsia is a significant complication during pregnancy and occurs in 3%–5% of pregnant women, with potentially fatal outcomes (1). Major complications occur to a higher extent in low- and middle-income countries due to the lack of preventive care and early recognition (1). Identifying biomarkers such as natriuretic peptides in hypertensive disorders of pregnancy suggests cardiac strain at the time of preeclampsia (2–4). Early identification of pregnant patients with increased cardiovascular risk may help stratification and guide therapeutic management to improve outcomes. Prior studies have shown that natriuretic peptide levels remain stable throughout pregnancy under normal conditions, whereas patients with preeclampsia have a significant increase throughout pregnancy (5, 6). Furthermore, elevated natriuretic peptide levels have been associated with increased adverse perinatal outcomes (7). While natriuretic peptide levels can vary depending on several factors such as age, a normal value within a healthy individual below the age of 50 is considered <100 pg/mL for b-type natriuretic peptide and <450 pg/mL for NT-proBNP due to its longer half-life (8).

Studies have described altered triglyceride and cholesterol levels and changes in Apo lipoproteins B and A1 as potential risk factors for preeclampsia (9). Furthermore, diabetes and obesity represent independent risk factors for developing preeclampsia (10). Changes in vascular physiology are key components of preeclampsia, and alterations in angiotensin-II, endothelin-1, and thromboxane A2 are associated with it. Furthermore, altered maternal mean arterial pressure and uterine artery resistance are highly predictive of preeclampsia development (11). Significant hemodynamic changes throughout pregnancy such as increased cardiac output and reduced systemic vascular resistance enable the transition to a high-volume, low-resistance circulation (11). Preeclampsia can present in numerous ways, from asymptomatic hypertensive patients to life-threatening placenta abruption or end-organ dysfunction such as peripartum cardiomyopathy (PPCM), a form of heart failure with reduced ejection fraction (HFrEF) that can occur during or shortly after pregnancy (12). The incidence of PPCM varies depending on the socioeconomic background of a country, ranging from 1:100 to 1 in several thousand. Furthermore, *in vitro* fertility and increased maternal age are expected to increase the risk of PPCM in the future (12). Early recognition, close monitoring, and delivery planning are key to reducing mortality for both mother and child (1).

## Materials and methods

In this observational report, we describe a series of four high-risk pregnant patients, aged 20–40 years, from different ethnic backgrounds and the role of natriuretic peptides as potential markers for the imminent development of preeclampsia and peripartum cardiomyopathy. Patients underwent regular transthoracic echocardiogram testing to assess ventricular function throughout pregnancy and regular measurement of natriuretic peptide levels. In this retrospective study, we analyzed changes in the left ventricular ejection fraction and left atrial volume and increases in natriuretic peptides in correlation with the development of preeclampsia. All patients within this case series had continuous access to health care and were compliant with their medication.

## Results

The principles outlined in the Declaration of Helsinki were followed in treating the described patients.

### Patient 1

**Figure 1:**
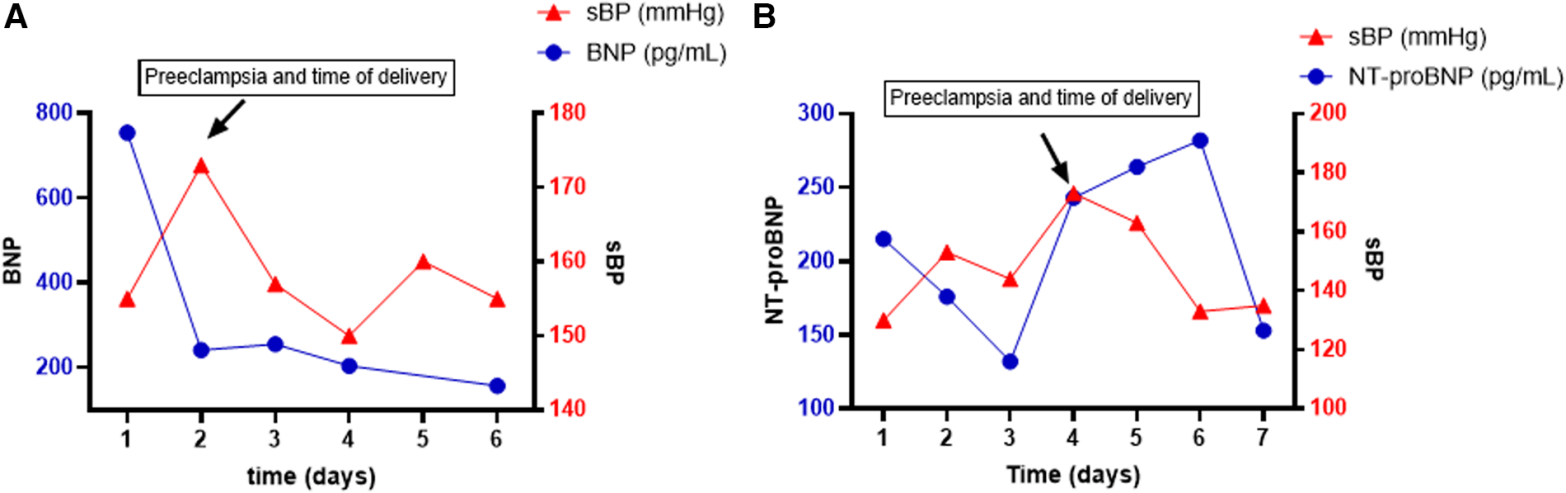
Time course (days) of systolic blood pressure (sBP) and BNP trend in patient 1 (**A**) and patient 2 (**B**). BNP is represented on the left *y*-axis in pg/mL, and sBP is represented on the right *y*-axis in mmHg.

A 20–25-year-old Black woman, body mass index (BMI) 40, G1P0 (Table 1) with a prior medical history (PMHx) of chronic hypertension, nephrotic syndrome, and anemia, was admitted to the hospital at 32w1d gestation due to recurrent hypertensive episodes (160 s/100 s). The patient developed superimposed severe preeclampsia reflected by an increase in creatinine (0.8 → 1.2 mg/dL) with a 24-h urine protein content of 10 g and a brain natriuretic peptide (BNP) content of 754 pg/mL (Figure 1A). She was admitted for continued observation and monitoring and initiated on nifedipine, hydralazine, and magnesium infusion for 24 h. A transthoracic echocardiogram (TTE) was obtained due to volume overload, which showed concentric hypertrophy of the left ventricle with an ejection fraction (EF) of 38% and a left atrial (LA) volume of 71 mL (normal <52 for woman, <58 for man), left atrial volume index (LAVI) 30 mL/m^2^ (normal <28 mL/m^2^). The patient underwent an urgent C-section due to a new diagnosis of preeclampsia-associated PPCM. Postdelivery, the patient was diuresed and discharged on nifedipine 120 mg daily, hydralazine 20 mg TID, and enalapril 2.5 mg. Six weeks postpartum, the patient’s LVEF remained reduced at 35% with an LVEDD of 57 mm (initially 47 mm). She was discharged on guideline-directed medical therapy (GDMT) for HFrEF and close follow-up in the combined cardio-obstetrics clinic. Her LA and LAVI normalized to 45 mL and 20 mL/m^2^, respectively.

**Table 1:** Demographic parameters.

	Patient 1	Patient 2	Patient 3	Patient 4
Age range (years)	20–25	30–35	30–35	35–40
Ethnicity	Black	White	Hispanic	Black
BMI	40	32.6	28.9	44
Pregnancy	G1P0	G2P1	G2P1	G2P1
Gestation week	32w1d	37w3d	Postpartum day 1	26w3d
Systolic blood pressure	174 mmHg	172 mmHg	wnl	162 mmHg
BNP	754pg/mL	N/A	N/A	N/A
NT-proBNP	N/A	243 pg/mL	330 pg/mL	81 pg/mL
EF	38%	35%	50%	N/A
LA volume	71 ml	70 ml	N/A	N/A

BMI, body mass index; BNP, brain natriuretic peptide; EF, ejection fraction; LA, left atrium; wnl, within normal limits; N/A, not applicable.

### Patient 2

A 30–35-year-old White woman, BMI 32.6, G2P1 (Table 1) with a PMHx of preeclampsia and chronic hypertension, was admitted for planned induction and delivery at 37w3d. The patient was evaluated in the emergency department (ED) 3 weeks prior due to the sudden onset of palpitations. In the ED, she was diagnosed with atrial fibrillation with rapid ventricular rate and was pharmacologically converted into sinus rhythm and initiated on metoprolol. A TTE at the time showed a dilated LV (LVEDD 71 mm) with an LVEF of 43%, an LA volume of 70 mL, and *de novo* severe aortic insufficiency (AI) without aortic root dilation. It is unclear whether her HFrEF resulted from her prior preeclampsia with PPCM or whether it is of valvular nature. Prior to admission, the patient had blood pressure values within normal limits. NT-proBNP was down trending at 132 pg/mL since the episode of atrial fibrillation. On the day of admission, her NT-proBNP increased to 243 pg/mL. Within a few hours of admission, she developed severe features of preeclampsia with a systolic blood pressure of 200 mmHg, and an emergent C-section was performed (Figure 1B). LVEF on the day of delivery was 35% with moderate to severe AI. After delivery, the NT-proBNP rapidly decreased and LVEF improved to 40%–45%. The patient was treated with hydralazine and furosemide postdelivery. Valvular replacement will be re-evaluated within 6 months postpartum.

### Patient 3

A 30–35-year-old Hispanic woman, BMI 28.9, G2P1 (Table 1) with a PMHx of preeclampsia with emergent preterm C-section, was admitted to the hospital. The patient developed chronic hypertension after her first pregnancy, together with HFrEF with an LVEF of 40%. With GDMT, the patient’s left ventricular function recovered, and she presented with an LVEF of 60%–65% at the time of her second pregnancy. The patient was admitted at 38w1d for a planned induction of labor. At the time of admission, the blood pressure was well controlled with labetalol 200 mg TID and hydralazine 25 mg TID, and the patient was continuously normotensive during labor. NT-proBNP was within the normal range, and serial TTE during her pregnancy showed a normal LVEF of 60%–65%. On postpartum day 1, the patient was treated with furosemide for increased peripheral edema. There was a significant increase in NT-proBNP from 65 to 330 pg/mL. A repeat TTE showed a reduced LVEF to a low normal 50%, and she was diagnosed with recurrent preeclampsia with PPCM. The patient was discharged on postpartum day 2 on labetalol and hydralazine and follow-up in the cardio-obstetrics clinic.

### Patient 4

A 35–40-year-old Black woman, BMI 44, G2P1 (Table 1) with PMHx of preeclampsia with preterm C-section at week 28 and chronic hypertension, was admitted to the hospital. The patient was seen in the cardio-obstetrics clinic at 26w3d, where she was diagnosed with intrauterine fetal death, likely due to recurrent preeclampsia. The patient had been hypertensive, with systolic BP in the 160s and tachycardia in the 115s for approximately 6 weeks. Her antihypertensive treatment prior to admission consisted of nifedipine 60 mg once daily. On admission, she was additionally diagnosed with hemolysis, elevated liver enzymes, and low platelet (HELLP) syndrome with the following laboratory values: fibrinogen 887 mg/dL, D-dimer 5,271 ng/mL, AST 86 IU/L, ALT 128 IU/L, and platelets 45 B/L. Nifedipine was increased to 90 mg/d, and labetalol 200 mg TID was initiated. NT-proBNP was 81pg/mL, possibly low due to increased BMI (44) and the late time frame of detection (13). TTE was planned for outpatient follow-up.

## Discussion

Preeclampsia is the leading cause of pregnancy-related morbidity and mortality (11). Importantly, women with a prior medical history of preeclampsia have poor long-term cardiovascular outcomes, and early recognition and medical management are crucial (11). Women in their 20s with a prior medical history of preeclampsia are predicted to have an inferior cardiovascular prognosis within the next 10 years postdelivery compared with women that are twice their age (11). Women with recurrent preeclampsia are at higher risk of developing chronic hypertension, ischemic heart disease, HF, and cerebrovascular events as well as hospitalization due to cardiovascular events up to 15 years postpregnancy (14). Immediate treatment of preeclampsia is delivery, leading to a resolution of preeclampsia-related symptoms, including reduction of stroke volume and cardiac output within a few days, whereas vascular resistance and mean arterial pressure will persist longer (15). In fact, approximately half of the patients with preeclampsia-induced hypertension will remain hypertensive 12 weeks postpartum (16).

**Figure 2:**
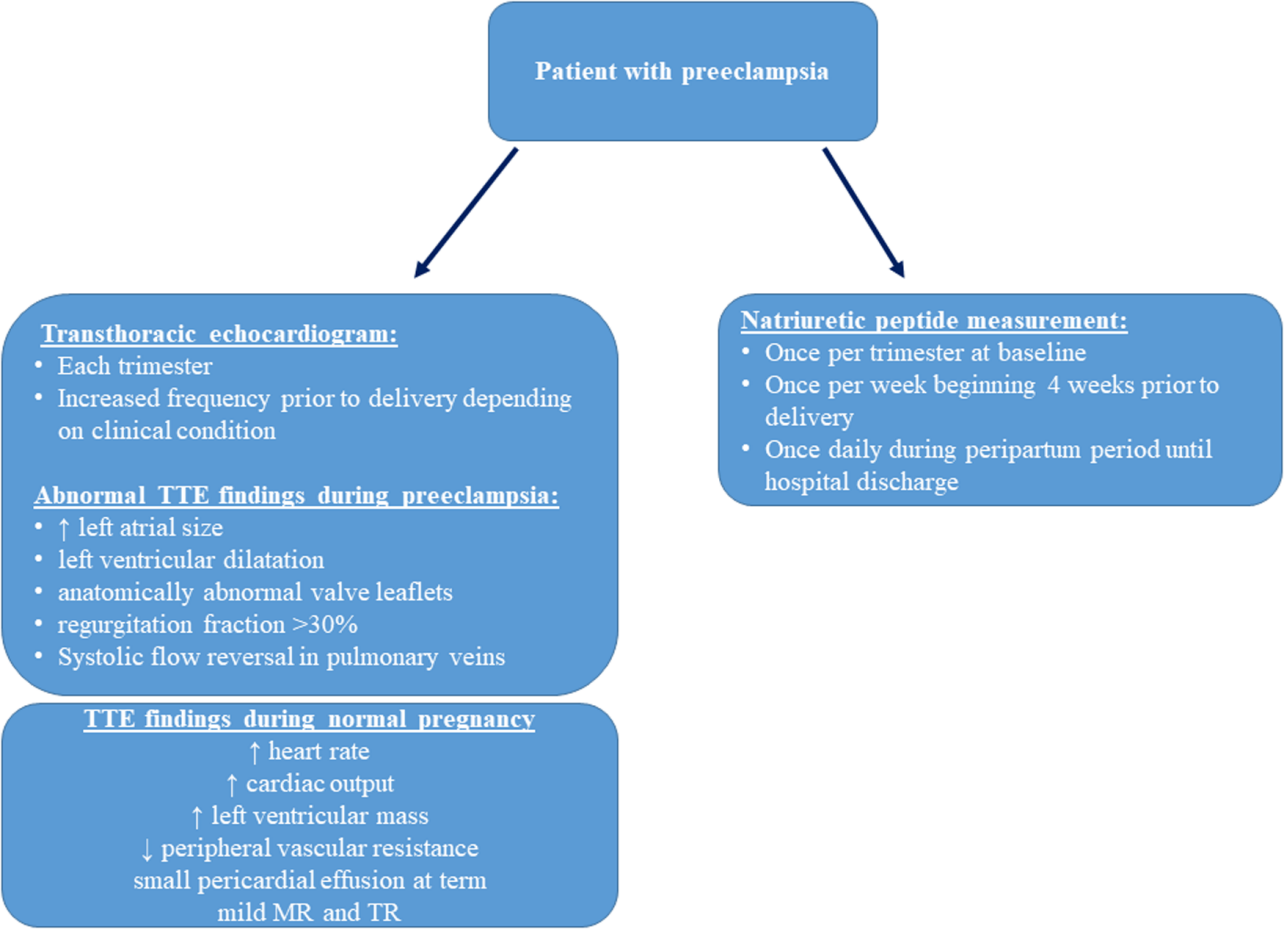
Proposed screening of high-risk patients for preeclampsia, physiologic hemodynamic transthoracic echocardiography (TTE) parameters in healthy pregnant patients, and abnormal TTE findings in preeclampsia (19).

The introduction of BNP and NT-ProBNP levels as diagnostic tools for patients with chronic hypertension allows us to identify cardiac complications like heart failure. A recent meta-analysis suggests that both BNP and NT-proBNP levels can assist in the diagnosis of HF and preeclampsia (17). BNP demonstrated better diagnostic accuracy compared to NT-proBNP (17). In this case series, we described four patients with preeclampsia and associated PPCM. The majority of patients developed chronic hypertension *after* being diagnosed with preeclampsia during their previous pregnancy, leading to a variety of clinical complications including fetal demise. Early recognition and treatment of preeclampsia with or without the development of PPCM is vital to prevent adverse maternal and fetal outcomes. Patients with a prior medical history of preeclampsia should be followed closely throughout pregnancy. Of note, an increase in BNP can precede the development of preeclampsia, as described above, and serial TTE and natriuretic peptide follow-up throughout pregnancy should be considered in high-risk patients (18). Interdisciplinary management of these patients in a cardio-obstetric clinic is highly recommended, and high-risk patients should be referred to centers offering such services. Most risk factors, symptoms, and long-term comorbidities of preeclampsia are of cardiovascular nature, and the development of new cardiovascular markers for early detection of preeclampsia is crucial to improve outcomes. With this case series, we propose an algorithm for managing high-risk patients for developing preeclampsia (Figure 2).

## Limitations

This study represents a case series of four patients with preeclampsia and therefore does not have a control group. Given that BNP levels can vary depending on factors such as creatinine, age, and obesity, the very small sample size needs to be considered. While previous studies have described elevated natriuretic peptide levels in patients with preeclampsia compared to healthy pregnancies, we aimed to describe our findings of a timely relationship between the increase in natriuretic peptide and the imminent development of preeclampsia. Little is known regarding the role of atrial natriuretic peptide (ANP) and endothelial development in preeclampsia. Few studies have investigated this field so far (20). While our institution currently does not evaluate routine ANP levels during pregnancy, further studies should also investigate its role in the development of preeclampsia.

## Data Availability

The raw data supporting the conclusions of this article will be made available by the authors without undue reservation.
